# Associations between Parental and Child Screen Time and Quality of the Home Environment: A Preliminary Investigation

**DOI:** 10.3390/ijerph17176207

**Published:** 2020-08-27

**Authors:** Parveen Attai, Jacqueline Szabat, Stephanie Anzman-Frasca, Kai Ling Kong

**Affiliations:** 1 Division of Behavioral Medicine, Department of Pediatrics, Jacobs School of Medicine and Biomedical Sciences, State University of New York at Buffalo, Buffalo, NY 14214, USA; parveena@buffalo.edu (P.A.); jmszabat@buffalo.edu (J.S.); safrasca@buffalo.edu (S.A.-F.); 2 Department of Epidemiology and Environmental Health, School of Public Health and Health Professions, State University of New York at Buffalo, Buffalo, NY 14214, USA; 3 Center for Ingestive Behavior Research, State University of New York at Buffalo, Buffalo, NY 14260, USA

**Keywords:** IT-HOME, screen use, screen media, parent-child interaction, home environment

## Abstract

(1) Background: The recommendation for screen use among preschool-aged children is ≤ 1 h per day. We aimed to assess the relationship between parental and child screen use and home environment characteristics. (2) Methods: Thirty-six 3–to-4-year-old healthy children were recruited. Parents reported their own and their child’s weekday and weekend daytime screen use. The child’s home environment and parent-child interactions were assessed using the Infant-Toddler Home Observation for Measurement of the Environment (IT-HOME). Analyses were run to identify relationships between parental and child screen use and the six subscales of the IT-HOME: Responsivity, Acceptance, Organization, Learning Materials, Involvement and Variety. (3) Results: Parents’ weekend screen use was correlated to parental responsivity and variety of people and events at home. These relationships remained significant after adjusting for maternal education and number of children at home (Responsivity β = 7.30 (95% CI: 1.75, 12.86), *p* = 0.012) and (Variety β = −2.45, (95% CI: −4.58, −0.31), *p* = 0.026). There was a trend level association between low child’s weekend screen use and high presence of learning materials. Other aspects of screen time were not associated with home environment characteristics. (4) Conclusions: Higher parental screen use predicted lower variety and greater parental responsivity, the latter of which was an unexpected finding. Administering the IT-HOME alongside a screen use questionnaire may offer the opportunity for a more comprehensive representation of home environments in today’s society. Future research can also clarify facets of parental screen use (e.g., co-viewing, timing) that are more vs. less likely to impact children.

## 1. Introduction

Early childhood is a critical time period for brain development [1]. During this time, a child’s brain is constructed to form the basis of sensorimotor, cognitive-language and social-emotional functioning [2]. Development in early life is multi-faceted and can be attributed to both biological and environmental factors, including genetics, culture and ethnicity, parenting, socio-economic status and home environment [3]. Environmental factors are modifiable; understanding and improving such factors could promote more positive developmental trajectories for children.

Parenting is one modifiable factor. In early childhood, parents and caregivers have a substantial amount of control over children’s early experiences. The impact of positive parenting strategies on children’s development has been demonstrated through intervention research, with benefits for children’s cognitive responsive behaviors [4] and emotional competence [5]. Aspects of positive parenting include being responsive to, accepting of and involved with the child while setting developmentally-appropriate limits [6].

Another important factor is the home environment, which should offer safe and age-appropriate stimulation for a child. The Infant Toddler-Home Observation for Measurement of the Environment (IT-HOME) characterizes a stimulating home environment as one that is organized, has learning materials and provides the child with a variety of experiences [6]. Home stimulation can benefit children later, helping them achieve better cognition and language skills. Nievar et al. (2014) reported that a stimulating home environment, regardless of financial state, better prepared children for school [7]. The home environment can also mediate the association between socio-economic status and literacy competence, as well as social functioning in preschool [8]. The effects of early home environment seem to persist; in a longitudinal study, results characterized children with more environmental stimulation at 4-years-old with a more advanced cortex at 17–to-19-years-old [9]; in this study, environmental stimulation was defined as degree of home organization, availability of learning materials and inclusion of various experiences in the child’s life.

The way researchers characterize children’s environments may need to be updated given historical changes, including the emergence of new technologies. Screen use is an area of recent research because it has become ubiquitous and dominating in the home environment. Common Sense Media (2017) reports that in households with a child under the age of 8, 98% have a TV set, 91% a computer, 98% a mobile device, 78% a tablet device and 69% a console video game player. Children are also beginning to own media devices; 43% of children 2–to-4-years-old have their own tablet device [10]. Children are introduced to screen media very early in life and then spend significant amounts of time interacting with screens. On average, children begin interacting with digital media at 4-months-old [11]. Common Sense Media also reports that children aged 2–to-4 are interacting with screens for about 2 h 39 min per day [10].

The American Academy of Pediatrics (AAP) suggests limiting screen use to one hour or less per day for children aged 2–to-5-years-old [12]. Their statement is based on literature that demonstrates the link between high screen use in preschoolers and negative effects, such as decreased self-regulation development and decreased parent-child interactions [13]. Recognizing that it is unlikely that all of the varied types of screen time in our modern environment have equal effects, the AAP also suggests that the hour of screen use be for high-quality programs with parents co-viewing [12], as this type of media consumption can be used to discuss values, solve problems and to enhance the connection between a parent and child [14], all of which support child development.

Because the screen-media surge has been fairly recent, the overall goal of this study was to look at the relationship between parental and child screen use and home environment characteristics (Responsivity, Acceptance, Organization, Learning Materials, Involvement and Variety) in preschool-aged children. This work fills a gap in the literature, as it is the first cross-sectional study to analyze parental and child screen use and their correlations with subscales from the IT-HOME, which is a valid, reliable and widely used measure that reflects aspects of both parenting and the home environment [6]. This work may inform future studies that utilize the IT-HOME to better understand the quality of home environment and screen time behaviors of young children.

## 2. Materials and Methods

### 2.1. Participants

Thirty-six 3–to-4-year-old healthy, singleton children from Buffalo, New York, were recruited to be part of this study. Participants were recruited in two cohorts. Cohort one (31 participants) was recruited between September 2017 and November 2018 and cohort two (five participants) was recruited between February and May 2019. Exclusion criteria included: mothers younger than 18 years of age at time of child’s birth, children with medical problems at birth or at present, children with any developmental condition or developmental delay and children who were a twin or a multiple. The participating parent provided written consent on behalf of their child. The University at Buffalo Institutional Review Board approved study procedures and participating parents provided informed consent for their participation.

### 2.2. Procedures

#### Overview

Interested families were screened either by an online screener or a telephone interview. Once determined as eligible, a laboratory visit was scheduled for the participating parent and child at a time of day when the child would be alert and willing to participate. The visit took place in the laboratory playroom, which was furnished with a sectional couch, a small bookshelf and a rug. Three cameras were installed at various locations and their display was visible from the control room, which was directly connected to the playroom. Four piles of toys (wooden alphabet blocks, a tool belt set, a bear family dress-up puzzle and a wooden cutting board with play food) were placed on top of the rug in a line. Upon arrival, the parent-child pair was brought into the playroom. A staff member went over the purpose of the visit, which was for the parent and child to spend time together “like they would at home”, while playing with the provided toys and then cleaning them up. Before starting, the staff member gave the parent a consent form to review and sign. During the ten minutes of playtime, the staff member was in the control room observing the parent-child interaction. For clean-up time, the staff member provided three bins labeled “Blocks”, “Tools” and “Other toys” and asked the child to clean up with parental assistance, if needed. The staff member returned to the control room to observe five minutes of clean-up time. The visit concluded with a parental interview, during which the child was given a different set of toys to play with.

### 2.3. Measures

#### 2.3.1. Demographics

Via questionnaire, parents self-reported mother’s age at time of study, maternal education level, race/ethnicity, marital status and parity. Parents also reported child’s race/ethnicity, sex, age and birth weight.

#### 2.3.2. Infant Toddler-Home Observation for Measurement of the Environment (IT-HOME)

The IT-HOME is a valid and reliable measure of the home environment for children aged 0–to-3 years old [6]. It focuses on the child’s experiences at home, i.e., how the child receives inputs from objects, events and transactions [15]. Though originally developed for administration in the home using 45 items, in this study it was administered in the lab environment using 43 items. The latter method has been used previously, showing high inter-rater reliability (intra-class correlations of 0.88 to 0.98) [16]. The 43 items were organized in six subscales to reflect different aspects of parenting and of the home: Responsivity (11 items) measures parental responsiveness to the child’s behavior, Acceptance (eight items) measures parental acceptance of the child and avoidance of child punishment, Organization (five items) measures the organization of the home, Learning Materials (eight items) measures the availability of learning and play materials, Involvement (six items) measures parental involvement and Variety (five items) measures the occurrence of stimulating experiences in the child’s life. Responsivity, Acceptance and Involvement reflect the quality of parenting and were scored through observation in play and clean-up time and also via parent interview. Organization, Learning Materials and Variety reflect the quality of the home environment and were scored during the interview only. Each item was scored with either a “1” (yes) or a “0” (no). Subscales were summed to obtain the total IT-HOME score (range 0–43).

#### 2.3.3. Reliability in Coding

One coder scored the IT-HOME for all thirty-six participants. Prior to scoring independently, a supervisor trained the coder to a 90% reliability threshold. After data collection for each cohort, the supervisor quality checked 30% of the data set to ensure that reliability was maintained. If the supervisor found any data to be below the threshold, the coder and supervisor resolved the codes together and those sub-threshold data were not considered as part of the 30% checked. In such cases, the supervisor quality checked additional data until 30% met the reliability threshold.

#### 2.3.4. Screen Use

At the end of the IT-HOME interview, the experimenter asked four additional questions, the purpose of which was to understand the family’s behavior around screen use. Screen use was defined as any time spent on screens like televisions, laptops, cell phones, iPads/tablets, or video game systems such as Xbox or PlayStation. The definition of screen use, the four questions and the answer bank were used in previous work [17]. The questions included: 1. Not including screen time for school/homework, how many hours does your child spend on screens on an average weekday? 2. Not including screen time for school/homework, how many hours does your child spend on screens on an average weekend day? 3. Not including screen time for work/school, how many hours do you spend on screens on an average weekday? 4. Not including screen time for work/school, how many hours do you spend on screens on an average weekend day? Participating parents reported on screen use for their child and for themselves on both weekdays and weekends, excluding screen time for school/homework or work/school, respectively. Each question used the same answer bank: “None”, “Less than one h per day”, “two–three h per day”, “four–six h per day”, or “seven or more h per day”. Response options were coded as 0, 0.5, 2.5, 5 and 7, respectively, which were adopted from previous work [17]. However, one difference is that “Less than 1 h per day” was coded as 0.5 instead of 1, which was used by Tang et al. (2018). Weekday and weekend day screen use were analysed separately because past literature supports a difference in screen use between these two kinds of days. Children of this age group typically have higher screen use on weekend days [18].

### 2.4. Data Analysis

First, distributions of the six subscales of IT-HOME (Responsivity, Acceptance, Organization, Learning Materials, Involvement, Variety) and child/parent screen time (weekday and weekends respectively) were examined. Skewed subscales (Responsivity, Acceptance, Organization, Involvement and Variety) were corrected using log transformations. Univariate Pearson product-moment correlation coefficients examined relationships between parental and child screen use and the six IT-HOME subscales. We repeated these analyses as multiple linear regression models, adjusting for significant univariate covariates: maternal education and number of children at home. IT-HOME subscales were treated as the dependent variables predicted by parent and child screen time variables. All analyses were performed using SYSTAT (Carlsbad, CA, USA).

## 3. Results

Descriptive statistics including means, standard deviations (SD) and ranges of maternal and child demographics are included in Table 1. Of the children, 58% were female and 83.3% were White. Almost all participating parents were mothers (97%) and were married (97.2%); most of them (77.8%) had completed two-year college or higher.

### 3.1. Participating Parent and Child Screen Use

Table 2 shows the distribution of screen time during weekdays and weekend days among parents and children. Of the children, 53% had more than one hour of screen use on weekdays, and 69% on weekend days. Of the parents, 69% had more than one hour of screen use on weekdays, and 83% on weekend days. There was a trend of increased screen use during weekend days for both the parent and the child. Parental and child screen use were significantly correlated on weekend days (r = 0.510, *p* = 0.001) and associated at a trend level on weekdays (r = 0.297, *p* = 0.079).

### 3.2. IT-HOME and Screen Use

Table 3 shows correlations between parental and child screen use and the six subscales of IT-HOME. Parents’ weekend screen use was positively correlated with their responsiveness to their child’s behavior (Responsivity), but negatively correlated with variety of stimulating experiences in the child’s life (Variety). After controlling for maternal education and number of children in the home, the associations remained significant for both Responsivity (β = 7.30 (95% CI: 1.75, 12.86), *p* = 0.012) and Variety (β = −2.45, (95% CI: −4.58, −0.31), *p* = 0.026). Parental screen use was not associated with any of the other IT-HOME subscales.

Overall, child screen use was not related to the quality of the home environment. Child screen use during the weekend was negatively and marginally correlated with availability of learning and play materials (r = −0.302, *p* = 0.074), but after controlling for maternal education and number of children at home, this relationship became non-significant (Learning Materials; β = −7.27, (95% CI: -16.54, 2.00), *p* = 0.120). Child screen use was not associated with the other IT-HOME subscales on weekdays or weekend days.

## 4. Discussion

This study aims to look at the relationships between parental and child screen use and the quality of the child’s home environment. In our sample, more than half of the children did not meet AAP’s screen time recommendation on weekdays, and the majority did not meet it on weekend days. To our knowledge, our study is novel in examining how parental and child screen use is related to the quality of a child’s home environment as determined by the IT-HOME. Parental screen use was linked with two IT-HOME subscales, Responsivity and Variety.

We observed an increase in screen use on weekend days compared to weekdays, for both the child and parent in our sample. This was consistent with previous literature [19], which found a trend level increase in the number of 5–to-6-year-old children who watched more than two hours of television on weekend days compared to weekdays. Screen use may increase on weekend days simply because children are home with more leisure time. During the week, time may be occupied with school or other activities. This is reflected in data by Child Trends (2019), who reports that 30% of preschool-aged children are enrolled in preschool [20]. Parents may be able to reduce their children’s weekend screen use by providing them with enriching alternatives. For example, a previous study implemented a reading program to reduce television-viewing time in third and fifth graders [21]. If alternative activities are available, children may choose to engage in those, rather than with screen media.

There was also a correlation between parental and child screen use, especially on the weekends. Parents who had higher screen use on weekend days were more likely to have children who demonstrated the same pattern. This was consistent with a previous study, which found that parents who had higher screen use had children who watched an additional half hour of television per day [22]. More recent work has concluded that parental screen time is the strongest predictor of screen time for children 0–to-8-years-old [23]. A possible explanation may be that children learn through watching their parents’ behaviors. Zimmerman and Rosenthal (1974) mention that a child can “acquire patterns which span a wide diversity of specific tasks through observation alone” [24]. Another possible explanation is greater co-viewing of media among parents and children on weekend days.

In the present study, parents who had higher screen use on weekend days were more responsive to their child, which was an unexpected finding. This finding contrasts with literature that shows an inverse relationship between parental screen use (across all screen media) and responsiveness toward children. Radesky et al. (2014) observed 55 parent-child dyads in a restaurant setting and found that parental mobile device use interfered with responding to their child’s cues for attention [25]. Kirkorian et al. (2009) also found increased background television to decrease the quantity and quality of parent-child interaction [26]. This phenomenon has been recently coined “technoference”, or interruptions in interpersonal interactions as a result of digital and mobile technology use [27]. However, research supports parents co-viewing high quality programming with their children because it can be utilized as a learning tool, especially if parents communicate with their children while viewing [28]. Increased communication is distinctly captured in the Responsivity subscale because eight out of the eleven items exist to evaluate the extent to which the parent verbalizes and responds to their child. If a portion of the parent screen use on weekend days is devoted to co-viewing with their child, then it is possible for increased parental screen use to manifest as increased parental responsiveness. It is also possible that parents higher on responsivity are using screens at times when their children are not in their direct care, such as when another caregiver is with their children, or when their children are asleep. Future research can elucidate the extent to which parent co-viewing practices and timing of parental screen use may explain the unexpected link between higher weekend screen time and parent responsivity.

While the aforementioned findings were inconsistent with previous work, an expected finding was that parental screen use on weekend days was negatively associated with the variety of stimulating experiences provided to the child. This association may reflect the fact that parents have less time to stimulate their child with various activities when they are engaged in screen use. Looking at the items corresponding to this IT-HOME subscale, Variety captures events unique for the child (e.g., “Parent reads stories to child at least 3 times weekly,” “Child eats at least one meal a day with mother and father,” “Family visits or receives visits from relatives once a month”). This finding aligns with discussions from previous work, with Lauricella et al. (2015) speculating that the home media technology environment is influenced by a variety of factors, one of which is parents’ own screen use [23]. It is possible that children may experience reduced variety in their life when their parent has higher screen use; this is a consideration to keep in mind if home environment assessments like the IT-HOME are administered in conjunction with questionnaires that can measure different aspects of familial screen use.

Prior to covariate adjustments, there was a marginally negative relationship between child’s weekend screen use and availability of learning materials, suggesting that children who have higher weekend screen use have fewer learning and play materials available to them at home. A possible explanation is that if children are occupied with screen-media devices, they will be less likely to show interest in tangible learning and play items. Research conducted by the Michael Cohen Group (2014) found that touch screens were the most frequent play type in children under 12, exceeding time spent playing with “traditional” toys such as puzzles or blocks [29]. This association was non-significant after controlling for maternal education and number of children in the home, suggesting that socio-demographics may influence both screen usage and availability of learning and play materials. Taking into consideration this marginal finding, as well as the null association between child screen use and other IT-HOME subscales, our findings suggest parental screen use to be more predictive of the home environment than child screen use. Future work is needed to develop a more nuanced understanding of this topic, given that the quality and modality of screen use may have positive, negative, or unknown impacts on young children [13].

The strengths of this study include observing parent and child interaction in a lab setting and having all visits conducted and all data coded by one trained and reliable staff member. One limitation of this study was that the IT-HOME, which was developed for use in 0–to-3-year-olds, was utilized to evaluate the home environments of 3 year olds whose ages ranged from 3.02 to 3.91 years. Yet research has shown that the factor structures of the IT-HOME and the Early-Childhood HOME (EC-HOME; [30]), designed for 3–to-6 year olds, are very similar, with researchers suggesting that they are measuring the same environmental attributes [31]. Another limitation was that screen use was collected using parent report. Parents may have under-reported screen use for themselves and for their children because of socially desirable responding [32]. We asked about child screen use excluding time for school/homework and parent screen use excluding time for work/school. Parents may have interpreted school, homework, or work screen use differently, which may have led to variability in the results. Information was also not collected on the specific type of screen media used by the parent or child. Recently, novel self-administered measures that collect information on various screen-media devices have been deemed valid and reliable in adults [33]. It has been more difficult to collect this information in preschool-aged children because in this age group parents typically report for their children. Webster et al. (2019) used questions from the National Health and Nutrition Examination Survey 2009–2010 questionnaire because of its similarity to other valid and reliable measures [34]. But, to our knowledge, a standard self-administered measure for preschoolers does not yet exist. Lastly, the sample size of our preliminary study is relatively small, which is particularly true of our second cohort (*n* = 5). We re-ran our analysis by excluding these five participants, and the results remained virtually the same.

The results of this pilot study are especially timely, as there has been a 36% increase in media consumption worldwide, due to the coronavirus outbreak [35]. Families are being kept home from school and work resulting in an increased screen usage for both educational and non-educational purposes. There has been increased interest in the implications of this screen use, and our study sheds light on potential implications for preschool-aged children. Finding that weekend screen use was most predictive of characteristics of the home environment is relevant in the context of COVID-19, as more days of the week may resemble weekends with closures of childcare centers and schools. For future directions, it may be worthwhile to administer the IT-HOME alongside a valid and reliable screen use questionnaire. Though developed 35 years ago, the IT-HOME remains as one of the most common valid and reliable instruments measuring the quality of a child’s home environment today. Its purpose is to measure the “quality and quantity of stimulation and support available to a child in the home environment” [36]. Yet the inventory contains no measures to reflect today’s media-saturated environment of young children. An IT-HOME administered with a screen use questionnaire could include measures about the presence and use of screen media at home. Specifically, the assessment could determine the types of screen-media devices being used, the time spent on different kinds of screen media (as they may have different attributes), the quality of media children are consuming and parent/caretaker screen-media behaviors such as co-viewing. These considerations can inform the development of multi-faceted screen use measures for preschoolers, as well as allow for more in-depth examinations between parent/child screen use and IT-HOME subscales.

## 5. Conclusions

Young children’s environments have drastically changed over the past few decades. The changes have been due, in part, to a rapidly evolving society. Many of the changes have been positive. Screen media, once initially praised as taking on the form of an “electronic babysitter”, are now restricted by parents and caregivers who are aware of their potential ramifications. However, in today’s complex media landscape, it is also likely to be true that not all screen use is equal in terms of its implications for children’s environments and development. Our data demonstrated that high screen use may reduce the variety of a home environment, which can hinder a child’s cognitive development and/or school readiness. Yet higher parental screen use was also linked with increased parent responsivity, raising questions about the role of timing of viewing, type of programming and the extent to which interactive co-viewing occurs. Maintaining an enriched home environment while incorporating appropriate co-viewing behaviors may be the way to navigate parenting in the prospective future. Future research is needed to shed light on this work and its implications for children in different types of family structures. We conclude that it may be beneficial to administer children’s home environment assessments such as the IT-HOME alongside a multifaceted screen use questionnaire, to reflect both the presence of screen media in the home and the many factors that contribute to the child’s screen time behaviors. It is important for us to consider the varying ways screen media can influence the lives of children and families to fully understand the implications for children’s development and to inform improved guidelines in our media-saturated environment.

## Figures and Tables

**Table 1:** Characteristics of eligible dyads (*n* = 36).

Characteristics	*n* (%)	Mean (SD ^a^)	Range
Maternal characteristics			
Age at enrollment, y		35.01 (5.38)	27.29–50.58
Education			
2-year college or higher	28 (77.8)		
Marital status			
Single	1 (2.8)		
Number of children		2.53 (1.36)	1–6
Race/ethnicity			
White, non-Hispanic	30 (83.3)		
Child characteristics			
Sex			
Male	15 (41.7)		
Age, y		3.51 (0.29)	3.02–3.91
Race/ethnicity			
White, non-Hispanic	30 (83.3)		
Birthweight, g		33.95 (4.23)	28.6–43.8
zBMI ^b^		0.73 (0.89)	−0.99–2.71

^a^ SD = standard deviation. ^b^ zBMI (Body Mass Index) calculated using WHO growth chart.

**Table 2:** Description of parental and child screen use (*n* = 36).

Screen Time (h)	*n* (%)
Participating parent	
Weekday screen use	
None	0 (0)
<1 h	11 (30.6)
2–3 h	22 (61.1)
4–6 h	3 (8.3)
Mean ± SD	2.10 ± 1.28
Weekend screen use	
None	0 (0)
<1 h	6 (16.7)
2– 3 h	25 (69.4)
4–6 h	5 (13.9)
Mean ± SD	2.51 ± 1.26
Child	
Weekday screen use	
None	0 (0)
<1 h	17 (47.2)
2–3 h	18 (50.0)
4–6 h	1 (2.8)
Mean ± SD	1.62 ± 1.15
Weekend screen use	
None	1 (2.8)
<1 h	10 (27.8)
2–3 h	18 (50.0)
4–6 h	7 (19.4)
Mean ± SD	2.36 ± 1.60

**Table 3:** Correlation between parental and child screen use and child’s home environment assessed by Infant-Toddler Home Observation for Measurement of the Environment (IT-HOME).

Subscales of IT-HOME						
Screen Time	Responsivity	Acceptance	Organization	Learning Materials	Involvement	Variety
Parent weekdays	−0.206	0.276	0.207	−0.092	0.142	0.110
Parent weekend	0.403 **	0.246	0.165	−0.156	−0.196	−0.376 **
Child weekdays	−0.189	−0.178	0.047	−0.176	−0.095	0.059
Child weekend	−0.248	−0.071	0.164	−0.302 *	0.182	0.194

** *p* < 0.05; * *p* < 0.10.
